# Association of presence/absence and on/off patterns of Helicobacter pylori oipA gene with peptic ulcer disease and gastric cancer risks: a meta-analysis

**DOI:** 10.1186/1471-2334-13-555

**Published:** 2013-11-20

**Authors:** Jingwei Liu, Caiyun He, Moye Chen, Zhenning Wang, Chengzhong Xing, Yuan Yuan

**Affiliations:** 1 Tumor Etiology and Screening Department of Cancer Institute and General Surgery, the First Affiliated Hospital of China Medical University, and Key Laboratory of Cancer Etiology and Prevention (China Medical University), Liaoning Provincial Education Department, Shenyang 110001, China

**Keywords:** *Helicobacter pylori*, *OipA*, Peptic ulcer disease, Gastric cancer

## Abstract

**Background:**

There are increasing studies examining the relationship between the status of *H. pylori oipA* gene and peptic ulcer disease (PUD) and gastric cancer (GC) but the results turn out to be controversial. We attempted to clarify whether *oipA* gene status is linked with PUD and/or GC risks.

**Methods:**

A systematically literature search was performed through four electronic databases. According to the specific inclusion and exclusion criteria, seven articles were ultimately available for the meta-analysis of *oipA* presence/absence with PUD and GC, and eleven articles were included for the meta-analysis of *oipA* on/off status with PUD and GC.

**Results:**

For the on/off functional status analysis of *oipA* gene, the “on” status showed significant associations with increased risks of PUD (OR = 3.97, 95% CI: 2.89, 5.45; P < 0.001) and GC (OR = 2.43, 95% CI: 1.45, 4.07; P = 0.001) compared with gastritis and functional dyspepsia controls. Results of the homogeneity test indicated different effects of *oipA* “on” status on PUD risk between children and adult subgroups and on GC risk between PCR-sequencing and immunoblot subgroups. For the presence/absence analysis of *oipA* gene, we found null association of the presence of *oipA* gene with the risks of PUD (OR = 1.93, 95% CI: 0.60, 6.25; P = 0.278) and GC (OR = 2.09, 95% CI: 0.51, 8.66; P = 0.308) compared with gastritis and functional dyspepsia controls.

**Conclusions:**

To be concluded, when *oipA* exists, the functional “on” status of this gene showed association with increased risks for PUD and GC compared with gastritis and FD controls. However, merely investigating the presence/absence of *oipA* would overlook the importance of its functional on/off status and would not be reliable to predict risks of PUD and GC. Further large-scale and well-designed studies concerning on/off status of *oipA* are required to confirm our meta-analysis results.

## Background

*Helicobacter pylori* (*H. pylori*) infection is the most important risk factor for the development of peptic ulcer disease (PUD) and gastric cancer (GC) [1]. Nearly half of the population worldwide harbors *H. pylori*, however, both GC and PUD occur in only a small portion of those carrying *H. pylori*[2]. Difference in the consequence of *H. pylori* infection could be at least partially explained by the high variability of colonizing *H. pylori* strains and host response to this microbe [3]. In addition to the host genetic susceptibility, sequence diversity of *H. pylori* virulence factor gene may affect the ability of these bacteria to colonize, persist, and/or induce severe diseases, and thus enable us to predict the consequences of their carriers [4].

*H. pylori* possesses various genes, some of which are virulence genes associated with gastroduodenal diseases. To date, several well-described virulence factors of *H. pylori* including CagA (cytotoxin-associated gene A product) and VacA (vacuolating cytotoxin A) have been linked to severe gastroduodenal diseases such as PUD and GC [5]. Apart from CagA and VacA, other virulence factors were discovered successively. A study reported by Yamaoka Y. et al. in 2000 and subsequent studies provided evidence that OipA (outer inflammatory protein A) is another important virulence factor in relation to the risks of PUD and GC [6]. The *oipA* gene (also known as HP0638) encoding for OipA protein is regulated by a switch (i.e. ‘on’ or ‘off’ patterns) by changing the number of CT dinucleotide repeats in the signal-peptide coding region of this gene. Yamaoka Y. et al’s report also unraveled that the on/off functional status of *oipA* is accommodated by slipped strand mispairing mechanism [4]. When there is 6, 9, (1 + 3), (2 + 3), (1 + 2), (1 + 1 + 1), (1 + 1 + 2) or other CT dinucleotide repeats that keep the peptide in frame, *oipA* gene is “on” status. Otherwise, the status is “off”, which is nonfunctional *oipA* gene [7]. This OipA protein appeared to be particularly important in inducing interleukin 8 (IL-8) secretion and facilitating the bacteria’s colonization in stomach [1].

Since the discovery of *oipA* in 2000 [6], the relationship between the status of *oipA* gene and risks of PUD and GC is of special interest. Two kinds of studies in relation to *oipA* gene were emerging, some of which investigated the presence/absence of *oipA*[8-14] while the others examined the functional on/off status of *oipA*[6,15-24]. However, the results of these studies turn out to be controversial mainly due to relatively small sample size. So far, no meta-analysis has been provided to evaluate the risks of PUD and GC in relation to *oipA* gene presence/absence or its on/off status. In the present study, aiming at elucidating the role of *oipA* genetic diversity in modulating the risks of PUD and/or GC, we performed a meta-analysis to investigate the association between the presence/absence and on/off status of *oipA* gene and PUD and GC risks.

## Methods

### Identification and eligibility of relevant studies

We systematically search the literatures of electronic databases including PubMed, Web of Science, Chinese National Knowledge Infrastructure (CNKI) and Wanfang database using the search terms of “OipA”, “outer inflammatory protein A” and “HP0638” in English databases and their corresponding Chinese terms in Chinese databases. When overlapping data exists, only the largest and latest study was selected for this meta-analysis. We contacted the author for specific raw data if the data presented in the article were not clear. The last search date was December 6, 2012.

Studies included in the present meta-analysis must meet the inclusion criteria as follows: observational studies concerning the association between the presence/absence (also defined as positive/negative) or on/off status of *oipA* gene and PUD or GC with control group of gastritis or functional dyspepsia (FD); studies published in English or Chinese; studies with *H. pylori*-positive cases and controls; studies with sufficient raw data for estimating odds ratios (OR) and their 95% confidence interval (CI). The main reasons for exclusion were reviews; animal experiments; case series; duplicate publications; no raw data after contacting the author; studies not in English or Chinese; and conference proceedings.

### Data extraction

Two authors (Jingwei Liu and Caiyun He) extracted the data from the inclusive studies independently. The conflict was resolved after discussion and consensus was finally reached on all of the extracted data. The following information was extracted from each study: first author, year of publication, ethnicity of the population, numbers of cases and controls, detection methods for *oipA* gene and *H. pylori* infection, primers for polymerase chain reaction (PCR), source of *H. pylori* isolates, adult or children of the study subjects, and disease diagnosis for control group.

### Evaluation of the validity of the included studies

We used the eight-item Newcastle–Ottawa scale (NOS) to assess the validity of the included studies [25], in which a study could be awarded a maximum of nine stars on items related to the selection (four stars), the comparability (two stars) and the exposure (three stars). The NOS scores of 1-3, 4-6, 7-9 were considered as low, intermediate and high quality, respectively.

### Statistical analysis

The statistical analysis was carried out by Stata software (Version 11.0; StataCorp, College Station, TX). The strength of association between the presence/absence or on/off status of *oipA* gene and PUD or GC was assessed by OR and their 95% CI. P value <0.05 was considered as statistically significant. Heterogeneity was measured by using Q statistic (P <0.10 indicates significant heterogeneity between studies) and I-squared (I^2^) value [26]. A fixed-effects model using Mantel-Haenszel method [27] was applied to calculate the pooled ORs when heterogeneity between studies was not significant. Otherwise, a random-effects model using DerSimonian and Laird method [28] was performed. Sensitivity analysis was performed to explore heterogeneity when significant heterogeneity was indicated. Subgroup analyses were performed to explore the effects of geographical region, GU or DU, adult or children, and *oipA* gene detection method. To compare the effect of *oipA* gene status on the risks of PUD and GC among different subgroups, the Breslow-Day test was used to assess the homogeneity of stratum-specific ORs across different subgroups. For Breslow-Day test, statistical significance was noted as P ≤ 0.10. In addition, publication bias were evaluated qualitatively by performing funnel plots and assessed quantitatively by Begg’s test [29] and Egger’s test [30], respectively. P value < 0.1 for Begg’s and Egger’s tests indicates significant publication bias.

## Results

### Study characteristics

**Figure 1:**
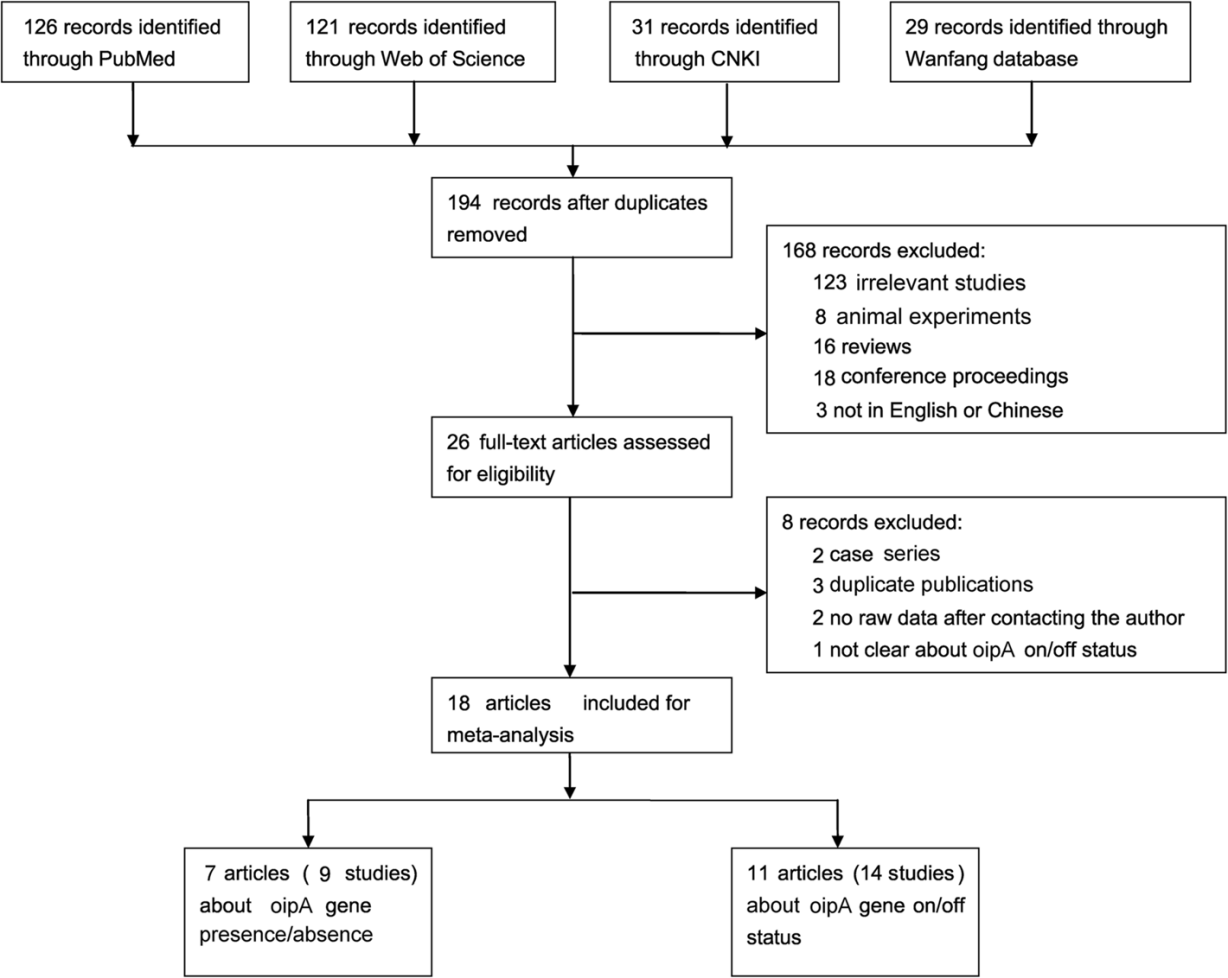
The flowchart of literature inclusion and exclusion.

This meta-analysis was organized according to the PRISMA statement (Additional file 1). A total of 194 potentially relevant records were found through four databases after duplicates removal; 176 articles were further excluded for main reasons of no relevance, in vitro or animal experiments, reviews, meeting abstract, data covered by other studies, and no raw data. Finally, 18 full-text articles with eligibility were included in this meta-analysis [6,8-24]. The flow chart of article selection was presented in Figure 1.

The main characteristics of the studies included in this meta-analysis were summarized in Table 1 and additional information of primary studies was showed in Additional file 2: Table S1 and Table S2. All the included studies were case–control designed. For meta-analysis of the on/off status of *oipA* gene, 11 articles met the inclusion criteria [6,15-24]. One article reported by Yamaoka, Y. in Japanese in 2000 found that the *oipA* gene status was 100% “on” in the cases and controls, which could not be analyzed in this meta-analysis. Therefore, 10 articles were finally eligible for the meta-analysis of the *oipA* on/off status. Among them, the functional on/off status of *oipA* was determined by the PCR-based sequencing with one exception of Yamaoka, Y et al’s study [21] in which immunoblotting was used to directly evaluate the protein expression of *oipA*; data were from three geographical regions including Asia, Europe and America; and one article [20] involved subjects of two different age groups (adults and children). For meta-analysis of the presence/absence of *oipA* gene, seven articles were ultimately eligible [8-14]. The presence/absence of *oipA* gene in the included articles was all detected by the PCR-based electrophoresis which could not reflect the functional status of *oipA*. Six articles were about Asian populations except one article involving Tunisian [9]. One article reported by Dabiri, H. et al. [11] involved study populations from three Asian countries. Considering a potential role of geographic location in altering the *oipA* gene status, data from different regions were treated as separate studies in the subgroup analysis. Data from different regions and different age groups were treated as separate studies in the subgroup analysis.

**Table 1:** **Characteristics of selected studies for *oipA *** **gene on/off and presence/absence status analysis**

**Author**	**Ethnicity**	**Year**	**Region**	**gastritis or FD**	**PUD**	**GU**	**DU**	**GC**
				***oipA*** **+/total (%)**^**d**^	***oipA*** **+/tota (%)**^**d**^	***oipA*** **+/total (%)**^**d**^	***oipA*** **+/total cases (%)**^**d**^	***oipA*** **+/total cases (%)**^**d**^
For *oipA* functional status (on/off) studies								
Markovska, R.	Bulgarian	2011	Europe	23/35(65.7%)	33/34(97.1%)	/	/	/
Oleastro, M.	Portuguese	2010	Europe	18/60(30.0%)	40/57(70.2%)	/	/	/
Schmidt, H. M.	Chinese	2010	Asia	45/52(86.5%)	14/16(87.5%)	/	14/16(87.5%)	19/22(86.4%)
Chiarini, A.	Italian	2009	Europe	17/21(81.0%)	7/10(70.0%)	/	/	/
LI, N.	Chinese	2009	Asia	66/86(76.7%)	12/12(100.0%)	12/12(100.0%)	/	2/2(100.0%)
Oleastro, M.	Portuguese^a^	2008	Europe	28/56(50.0%)	34/50(68.0%)	/	/	/
	Portuguese^b^	2008	Europe	17/53(32.1%)	26/31(83.9%)	/	/	/
Yamaoka, Y.	Colombian	2006	America	24/40(60.0%)	36/40(90.0%)	/	36/40(90.0%)	36/40(90.0%)
	American	2006	America	25/40(62.5%)	34/40(85.0%)	/	34/40(85.0%)	/
de Jonge, R.	Dutch	2004	Europe	18/29(62.1%)	39/49(79.6%)	16/21(76.2%)	23/28(82.1%)	8/9(88.9%)
Zambon, C. F.	Itilian	2003	Europe	15/31(48.4%)	14/19(73.7%)	/	/	/
Yamaoka, Y.	American	2002	America	25/40(62.5%)	37/41(90.2%)	/	37/41(90.2%)	20/30(66.7%)
	Colombian	2002	America	26/40(65.0%)	36/40(90.0%)	/	36/40(90.0%)	34/41(82.9%)
Yamaoka, Y.	Japanese	2000	Asia	40/40(100.0%)	40/40(100.0%)	/	40/40(100.0%)	/
For *oipA* gene (presence/absence) studies								
Ji, C. W.	Chinese	2011	Asia	26/46(56.5%)	45/58(77.6%)	45/58(77.6%)	/	33/43(76.7%)
Ben, M. K.	Tunisian	2010	Africa	186/195(95.4%)	63/78(80.8%)	/	/	/
Xie, J.	Chinese	2010	Asia	16/51(31.4%)	12/39(30.8%)	6/19(31.6%)	6/20(30.0%)	19/27(70.4%)
Dabiri, H.	Persians	2009	Asia	36/57(63.2%)	2/13(15.4%)	/	/	0/4(0.0%)
	Turkish	2009	Asia	7/21(33.3%)	3/5(60.0%)	/	/	1/7(14.3%)
	Kurds, etc.^c^	2009	Asia	8/13(61.5%)	4/4(100.0%)	/	/	/
Zhou, M.	Chinese	2009	Asia	21/40(52.5%)	37/40(92.5%)	/	37/40(92.5%)	18/20(90.0%)
Salih, B. A.	Turkish	2007	Asia	17/21(81.0%)	13/14(92.9%)	/	/	/
Zhang, J.	Chinese	2004	Asia	11/42(26.2%)	16/16(100.0%)	16/16(100.0%)	/	/

^a^, for adult study population; ^b^, for children study population; ^c^, Kurds, Lurs, Afghanis, Arabs; ^d^, for *oipA* gene presence/absence studies, *oipA*(+) means *oipA* gene positive, while for *oipA* functional status on/off studies, *oipA*(+) means *oipA* gene “on” status.

*Abbreviations*: *FD* functional dyspepsia, *PUD* peptic ulcer disease, *GU* gastric ulcer, *DU* duodenal ulcer, *GC* gastric cancer.

The NOS results indicated that all the included studies were at an intermediate level of quality with scores ranging from 4 to 6, because some studies did not provide specific selection criteria of control group and most studies did not fully consider the control factor for the comparability of cases and controls such as age and sex. Detailed results for NOS quality assessment were summarized in Additional file 2: Table S3.

### Association between oipA on/off status and PUD and GC

**Figure 2:**
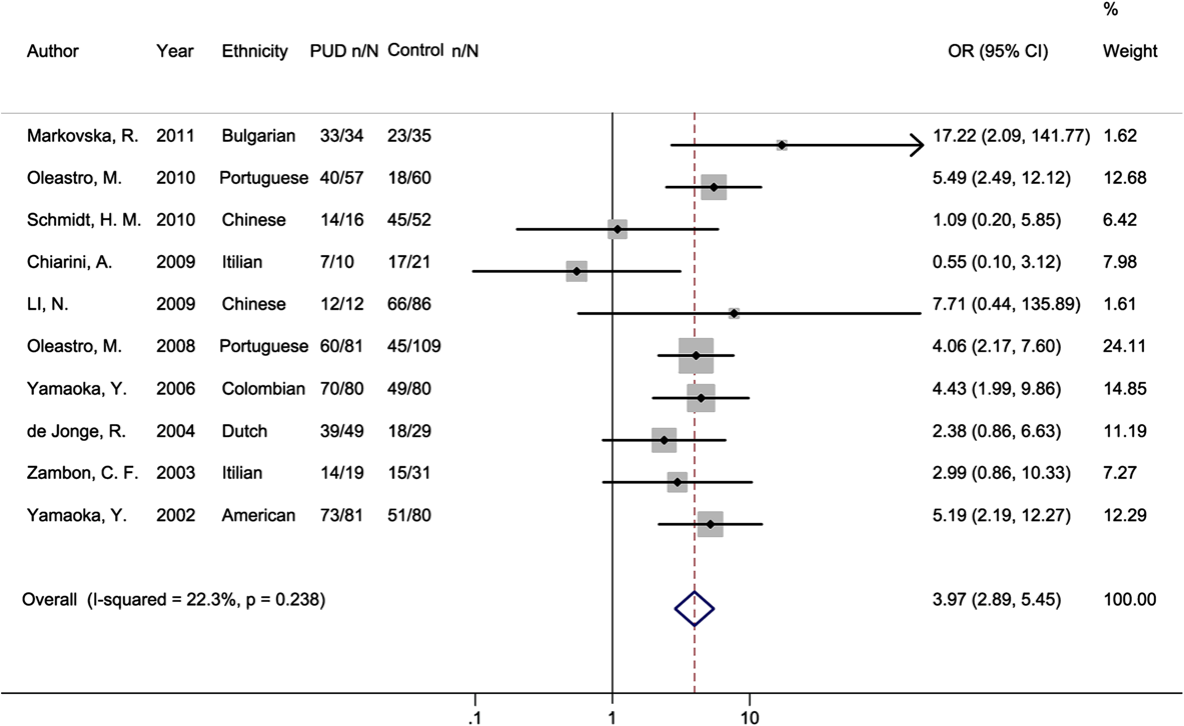
**Forest plot for the association between *oipA *** **“on” status and PUD risk.**

In the pooled estimate for PUD, we observed that *oipA* “on” status was significant associated with an increased its overall risk compared with gastritis and FD controls (OR = 3.97, 95% CI: 2.89, 5.45; P < 0.001, Table 2 and Figure 2). Meanwhile, no significant heterogeneity existed among studies (I^2^ = 22.30%, P = 0.238). Subgroup analysis was then performed to investigate the effects of geographical region, detection method of *oipA* gene and adult or children of the studied population. Consistent increased risks for PUD development were found in all subgroup analyses, with ORs ranging from 2.42 to 7.03. However, the result in Asia subgroup did not reach statistical significance (P = 0.208). Result of Breslow-Day test indicated no significantly different effects of *oipA* “on” status on PUD risk among Asia/Europe/America subgroups (P = 0.681, Table 2). The OR for PUD risk was higher in children subgroup than that in adult subgroup, and meanwhile, the effect difference reached statistical significance (for Breslow-Day test: P = 0.014). For DU and GU separately, we observed that *oipA* “on” status was significant associated with an increased risk of DU compared with gastritis and FD controls (OR = 3.83, 95% CI: 2.32, 6.34; P < 0.001), but the association between *oipA* “on” status and GU risk did not reach statistical significance (OR = 2.85, 95% CI: 0.94, 8.69; P = 0.065).

**Table 2:** **Meta-analysis results for association between *oipA *** **on/off and presence/absence status and PUD and GC**

**Variable**	**No. of studies**	**No. of cases/controls**	**OR (95% CI)**	**P value**	**I**^**2**^	**P**_**het**_^**a**^	**P**_**B-D test**_^**b**^
For *oipA* on/off studies							
PUD vs. gastritis/FD							
All	10	439/583	**3.97 (2.89,5.45)**	**<0.001**	22.30%	0.238	
Region							0.681
Europe	7	250/285	**3.75 (2.54,5.53)**	**<0.001**	57.20%	0.029	
Asia	2	28/138	2.42 (0.61,9.55)	0.208	32.90%	0.222	
America	4	161/160	**4.77 (2.65,8.56)**	**<0.001**	0.00%	0.902	
Detection technique							0.376
PCR based-sequencing	11	359/503	**3.83 (2.71,5.40)**	**<0.001**	41.10%	0.075	
Immunoblot	2	80/80	**4.42 (1.98,9.83)**	**<0.001**	0.00%	0.492	
Age							**0.014**
Adult	11	351/470	**3.26 (2.26,4.69)**	**<0.001**	16.90%	0.282	
Children	2	88/113	**7.03 (3.71,13.34)**	**<0.001**	0.00%	0.319	
GU vs. gastritis/FD	2	33/115	2.85 (0.94,8.69)	0.065	0.00%	0.368	
DU vs. gastritis/FD	6	205/241	**3.83 2.32,6.34)**	**<0.001**	0.00%	0.628	
GC vs. gastritis/FD							
All	5	144/287	**2.43 (1.45,4.07)**	**0.001**	16.80%	0.308	
Region							0.697
Europe	1	9/29	4.89 (0.54,44.57)	0.159	/	/	
Asia	2	24/138	1.08 (0.29,3.99)	0.909	0.00%	0.796	
America	3	111/120	**2.47 (1.36,4.51)**	**0.003**	51.30%	0.128	
Detection technique							**0.034**
PCR based-sequencing	5	104/247	1.75 (0.96,3.18)	0.066	0.00%	0.634	
Immunoblot	1	40/40	**6.00 (1.79,20.15)**	**0.004**	/	/	
For *oipA* presence/absence studies							
PUD vs. gastritis/FD							
All	7	267/486	1.93 (0.60,6.25)	0.278	85.70%	<0.001	
Region							**<0.001**
Asia	8	189/291	2.64 (0.85,8.20)	0.092	76.40%	<0.001	
Africa	1	78/195	**0.20 (0.08,0.49)**	**<0.001**	/	/	
GU vs. gastritis/FD	3	93/139	3.55 (0.71,17.77)	0.123	76.00%	0.016	
DU vs. gastritis/FD	2	60/91	3.15 (0.28,35.66)	0.354	87.10%	0.005	
GC vs. gastritis/FD	4	101/215	2.09 (0.51,8.66)	0.308	79.10%	0.002	

The statistically significant results were highlighted in bold.

^a^, P value for heterogeneity test; ^b^, P value for Breslow-Day test.

*Abbreviations*: *FD* functional dyspepsia, *PUD* peptic ulcer disease, *GU* gastric ulcer, *DU* duodenal ulcer, *GC* gastric cancer.

**Figure 3:**
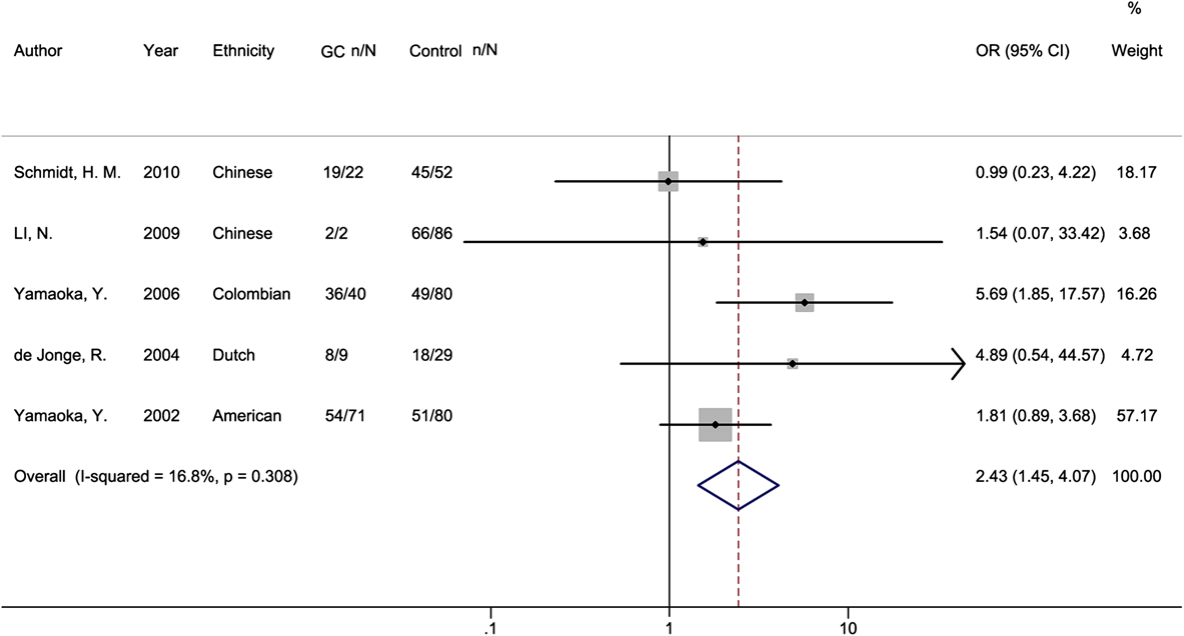
**Forest plot for the association between *oipA *** **“on” status and GC risk.**

In the pooled estimate for GC, the o*ipA* “on” status was significantly associated with its overall risk compared with gastritis and FD controls (OR = 2.43, 95% CI: 1.45, 4.07; P = 0.001, Table 2 and Figure 3). No significant heterogeneity existed among studies (I^2^ = 16.80%, P = 0.380). In the subgroup analysis according to geographical region, a statistically increased risk for GC was observed in the America subgroup (OR = 2.47, 95% CI: 1.36, 4.51; P = 0.003) and no significant association was found for Europe and Asia subgroups. However, result of Breslow-Day test indicated no significant different effects of *oipA* “on” status on GC risk among different regions. Whereas in the subgroup analysis of detection method, only the association in immunblot subgroup reached significance (OR = 6.00, 95% CI: 1.79, 20.15; P = 0.004), and the Breslow-Day test indicated a significant difference (P = 0.034) among the sequencing/immunoblot subgroups in the ORs for *oipA* “on” status with GC risk.

### Association between oipA gene presence/absence and PUD and GC

**Figure 4:**
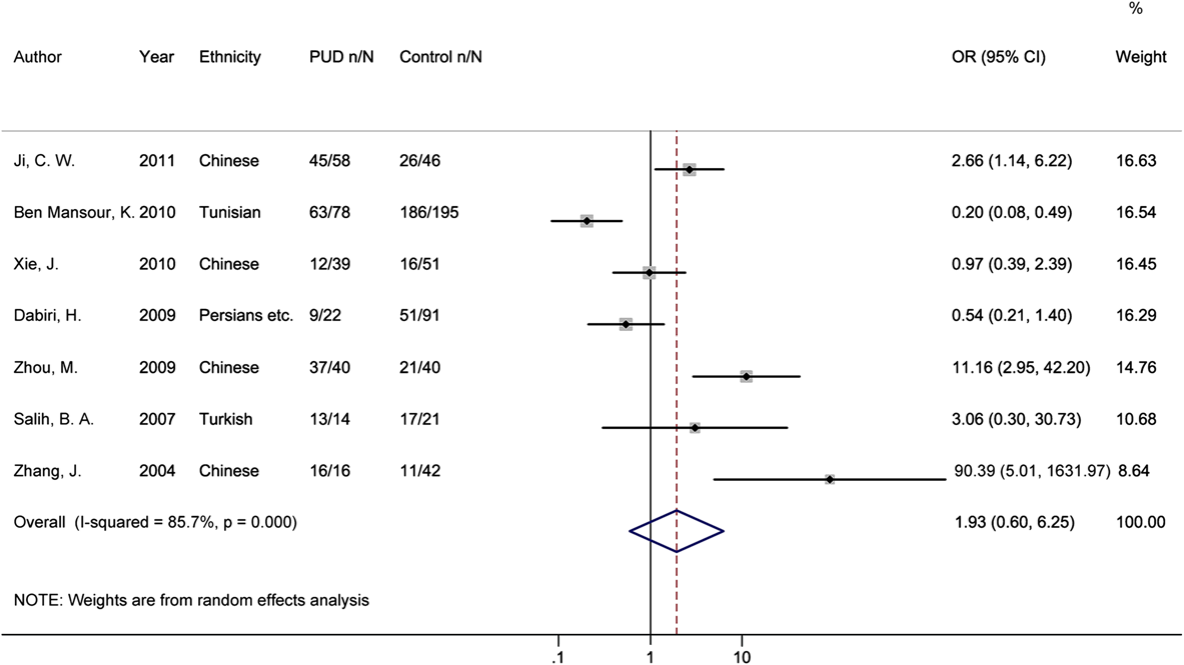
**Forest plot for the association between the presence of *oipA *** **gene and PUD risk.**

In the pooled estimate for PUD, the presence of *oipA* gene was not statistically associated with its overall risk compared with gastritis and FD controls (OR = 1.93, 95% CI: 0.60, 6.25; P = 0.278, Table 2 and Figure 4). However, it demonstrated a significant heterogeneity among studies (I^2^ = 85.70%, P < 0.001). To explore the source of heterogeneity, sensitivity analysis was performed. After the omitting of the most obvious outlier study with an OR of 90.39 (Figure 4) [14], significant heterogeneity still remained (I^2^ = 84.8%, P < 0.001) and the conclusion did not change. Subgroup differences and study design or quality also could not explain the source of heterogeneity. In subgroup analysis of different region, Africa subgroup analysis with only one article showed that the presence of *oipA* gene was statistically associated with a decreased risk of PUD compared with gastritis and FD controls (OR = 0.20, 95% CI: 0.08, 0.49, P < 0.001). Moreover, the Breslow-Day test did indicate a significant difference between Asia and Africa subgroups in the ORs for the presence of *oipA* gene (P < 0.001). For DU and GU separately, the presence of *oipA* gene was also not related with the risks of both diseases.

**Figure 5:**
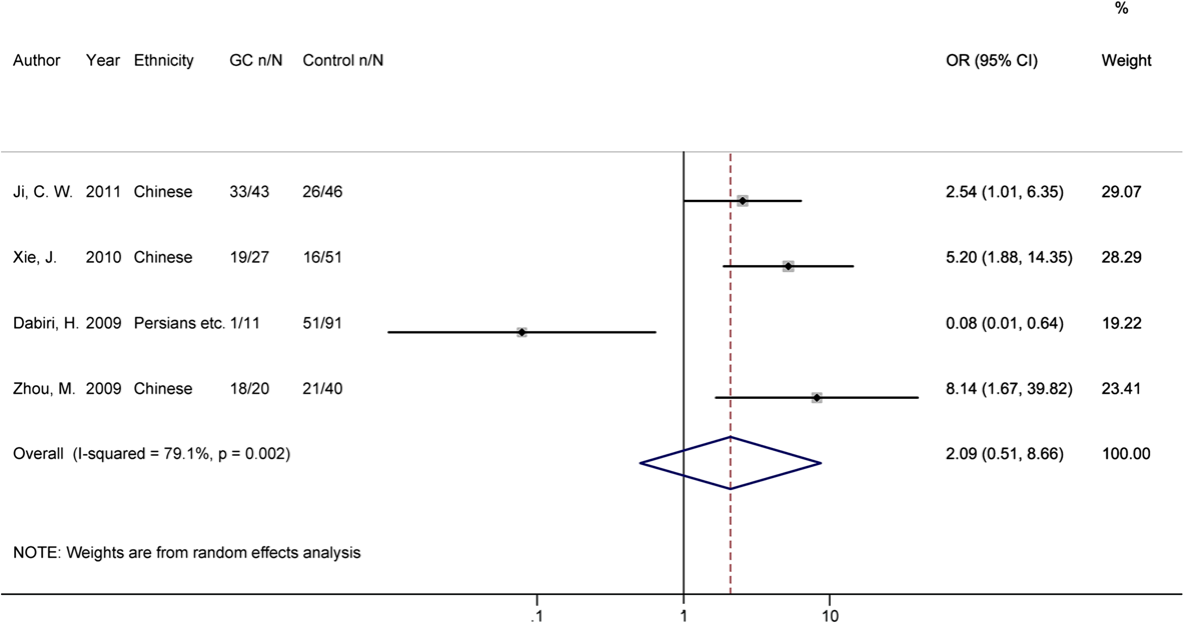
**Forest plot for the association between the presence of *oipA *** **gene and GC risk.**

In the pooled estimate for GC, we also observed no significant association of *oipA* gene positive with its overall risk compared with gastritis and FD controls (OR = 2.09, 95% CI: 0.51, 8.66; P = 0.308, Table 2 and Figure 5). And meanwhile, significant heterogeneity was indicated among studies (I^2^ = 79.1%, P = 0.002), thus sensitivity analysis was performed. The outlier study seemed to be the one carried out by Dabiri, H. et al. with an OR of 0.08 (Figure 5) [11]. After removing this data, the heterogeneity was no more significant (I^2^ = 0.00%, P = 0.372), and the presence of *oipA* gene was associated with increased GC risk in the remaining studies (OR = 4.11, 95% CI: 2.22, 7.62; P < 0.001). All the studies for GC risk used the PCR-electrophoresis method to detect the *oipA* presence/absence status and the studied populations all came from Asian region. Therefore, no subgroup analysis was performed for the presence/ absence status of *oipA* gene with GC risk.

### Publication bias

Funnel plots that qualitatively evaluated the publication bias of association between *oipA* presence/absence or on/off status and PUD were presented in Additional file 3: Figure S1. The Begg’s test and Egger’s test were performed to quantitatively evaluate the publication bias of the studies. No significant publication bias was observed for meta-analyses of presence/absence or on/off patterns of *oipA* gene. The results for publication bias test were showed in Table 3.

**Table 3:** Publication bias

**Variable**	**Begg’s test**		**Egger’s test**	
	**z value**	**P value**^**a**^	**t value**	**P value**^**a**^
For *oipA* on/off status studies				
PUD vs. gastritis/FD	−0.63	0.531	−0.77	0.465
GC vs. gastritis/FD	0.00	1.000	0.23	0.832
For *oipA* presence/absence studies				
PUD vs. gastritis/FD	1.05	0.293	1.64	0.163
GC vs. gastritis/FD	−0.68	0.497	−0.93	0.452

^a^, P value < 0.1 was considered as significant publication bias.

*Abbreviations*: *FD* functional dyspepsia, *PUD* peptic ulcer disease, *GC* gastric cancer.

## Discussion

Results of studies regarding the relationship of *H. pylori oipA* gene with PUD and GC risks turn out to be controversial [9,11,12,14,16-18,21]. To our knowledge, this is the first meta-analysis evaluating the association between *oipA* gene status and PUD and GC. By performing the current meta-analysis, we found that when *oipA* gene exists, the *oipA* “on” status was associated with increased risks of PUD and GC compared with gastritis and FD controls. Null association was found between the presence of *oipA* gene and PUD or GC risks.

For the pooled analysis of *oipA* gene on/off status, we found association of *oipA* gene “on” status with increased overall risk of PUD compared with gastritis and FD controls, and no significant heterogeneity among studies was observed. Consistently, increased risks for PUD were observed in subgroup analyses although associations in Asia and GU subgroups did not reach statistical significance, which possibly due to small sample size of the PUD cases in Asia and GU subgroups (28 and 33 cases respectively). The potential influence of geographical region, age and detection method on the association strengthen between *oipA* “on” status and PUD risk was further clarified by homogeneity test. Observations indicated that children infected by *H. pylori* with *oipA* “on” status have a higher PUD risk than adults. One of the possible reasons explaining this phenomenon is that the gastric microenvironment of children may be more suitable for *H. pylori* with *oipA* “on” status to induce PUD. Another possible reason is the relatively low defense ability of children against pathogenic factor. Nevertheless, this different effect of *oipA* “on” status between children and adults was obtained with small groups of strains, which therefore requires future validation.

The pooled estimate also demonstrated that the *oipA* “on” status was associated with an increased risk of GC compared with gastritis and FD controls, and no significant heterogeneity among studies was observed. Subgroup analyses indicated that *oipA* “on” status showed a consistent tendency toward increasing the risk of GC development although Europe, Asia, PCR-sequencing subgroups did not reach statistical significance. However, the test for homogeneities between subgroups indicated no significant difference for Europe/Asia/America subgroups. Statistical different effect was only indicated between PCR-sequencing and immunoblot subgroups. Although the PCR-based sequencing determines the functional status of *oipA* gene by detecting the number of CT repeats in its signal-peptide region, this method could not guarantee the expression of OipA protein. The high degree of genetic diversity of *oipA* gene may also complicate the interpretation of PCR based methods and may possibly result in an underestimation of the frequency of the functional status of the *oipA* gene.

Some mechanism studies may partially explain the association between *oipA* “on” status with PUD and GC. In 2000, by gene knockout models, Yamaoka et al. initially linked *oipA* gene “on” status with increased IL-8 production in gastric cancer cells [6]. Similar effects were detected in Straubinger et al’s study using cat models [31]. Subsequently, by challenging the volunteers with a *cagA* negative, *oipA* functional “on” strain of *H. pylori*, Graham et al. confirmed the role of *oipA* “on” status in inducing IL-8 levels in human. This author reported that the IL-8 levels in gastric mucosa increased even up to 20-fold by two weeks after inoculation [32]. Yamaoka et al. further unraveled that OipA was necessary for full activation of the IL-8 promoter and acted via the STAT1-IRF1-ISRE pathway [33]. Importantly, the IL-8 is one of the most essential proinflammatory factors, which acts as a potent chemoattractant and activator of neutrophils [34]. It has been suggested that IL-8 is closely linked with the tumorigenesis, angiogenesis and intracellular adhesion of cancer [35]. Therefore, it is tempting to speculate that the association of *oipA* “on” status with increased PUD and GC risks may be, at least in part, explained by a role in inducing IL-8 secretion.

It is worth noting that the detection of the presence/absence of *oipA* gene could not reflect the specific functional status of this gene, since the signal-peptide region of *oipA* gene varies significantly among *H. pylori* strains. Merely assessing the presence/absence of *oipA* gene would result in an underestimation of the influence of the functional status of *oipA* gene on PUD and GC development. Therefore, more caution should be taken when link the presence of *oipA* gene with phenotypic risk regardless of its functional status. The investigators that only examined the presence/absence of *oipA* gene may overlook the importance of the on/off status of *oipA* or could not perform the sequencing. The synthesis of the data in the present meta-analysis also suggested that only studying the “presence/absence” of *oipA* gene provided insufficient evidence to investigate the exact role of *H. pylori oipA* gene in gastrointestinal diseases. Normally, investigators should sequence *oipA* gene and take the functional on/off status of *oipA* into consideration.

The prevention and treatment for PUD and GC cause significant financial burdens around the world [36,37]. Because *H. pylori* is one important cause for PUD and GC, the identification of specific type of *H. pylori* isolates associated with PUD and GC risks would significantly reduce the costs of the screening and prevention for PUD and GC. The OR for PUD was found to be higher than that of GC (3.97 vs. 2.43) in this meta-analysis. The predictive role of *oipA* functional status in risks of PUD and GC may differ because of their distinct pathogenic mechanisms. However, we could not definitely conclude that the *oipA* “on” status is more closely linked with PUD than GC by numerically judging the OR values based on the currently-limited study samples. It is anticipated that *oipA* “on” status would be a promising indicator for *H. pylori* infected patients with increased risk for PUD or GC in the future.

We are aware that this meta-analysis has its own limitations. First, only articles in English or Chinese were selected. And we searched four databases without referring to other databases like EMBASE, which may result in selection bias. Second, the quality assessment of NOS indicated that most studies were at an intermediate level of quality mainly due to not matching for age or gender. In addition, the control group mixed the gastritis and FD, and some studies did not clearly describe that the control group excluded other kinds of diseases, which may result in an underestimation of the effect of *oipA* gene. Third, the sample size is not sufficiently large which may partially due to the limitation of the present laboratory technique for the isolation and cultivation of *H. pylori* isolates. Besides, numbers of included studies were relatively small, so the power for publication bias test was relatively low. Fourth, most studies were from Asia, thus the generalizability of our conclusion was limited. Fifth, significant heterogeneities were indicated for some comparisons particularly in presence/absence analysis, which could not be explained by subgroup analyses or sensitivity analyses. Moreover, the limited number of included studies precluded us from performing meta-regression to further explore the source of heterogeneity. Sixth, other important data such as age, gender, family history, status of other virulence factors and environment factors were not available to investigate the interaction between *oipA* gene status and these factors. The combined effect of *oipA* and other virulent factors should also be studied in future study.

## Conclusions

To be concluded, when *oipA* exists, the functional “on” status of this gene showed association with increased risks for PUD and GC compared with gastritis and FD controls. However, merely investigating the presence/absence of *oipA* would overlook the importance of its functional on/off status and would not be reliable to predict risks of PUD and GC. Further large-scale and well-designed studies concerning on/off status of *oipA* are required to confirm our meta-analysis results.

## Competing interests

The authors declare that they have no competing interests.

## Authors’ contributions

JL and CH performed statistical analysis, data interpretation and wrote the paper. MC and ZW analyzed the data and revised the manuscript. CX and YY conceived and designed this study and revised the manuscript. All authors read and approved the final manuscript.

## Pre-publication history

The pre-publication history for this paper can be accessed here:

http://www.biomedcentral.com/1471-2334/13/555/prepub

## Supplementary Material

Additional file 1 PRISMA checklist. Click here for file

Additional file 2: Table S1 Primers of PCR for *oipA* gene presence/absence and on/off status detection. Table S2. Detailed information of the included studies. **Table S3.** Results of Newcastle – Ottawa scale (NOS) assessment for the included studies. Click here for file

Additional file 3: Figure S1 Funnel plots of *oipA* gene presence/absence and *oipA* gene on/off status and PUD A, funnel plot for studies of association between *oipA* gene presence/absence and PUD; B, funnel plot for studies of association between *oipA* gene on/off status and PUD. Click here for file
