# Supplementary material for: Association of presence/absence and on/off patterns of Helicobacter pylori oipA gene with peptic ulcer disease and gastric cancer risks: a meta-analysis
Source: BMC Infect Dis. 2013 Nov 20;13:555. doi: 10.1186/1471-2334-13-555 (PMC4225565; doi:10.1186/1471-2334-13-555)
Supplement: Additional file 2: Table S1 — Primers of PCR for oipA gene presence/absence and on/off status detection. Table S2. Detailed information of the included studies. Table S3. Results of Newcastle – Ottawa scale (NOS) assessment for the included studies. [file 1471-2334-13-555-S2.doc]

Supplementary Table 1. Primers of PCR for *oipA* gene presence/absence and on/off status detection

| Author | Population | Year | Forward primer | Reverse primer | | product |
| --- | --- | --- | --- | --- | --- | --- |
| For *oipA* functional status (on/off) studies | | |  |  | |  |
| Markovska, R. | Bulgarian | 2011 | 5′-GTTTTTGATGCATGGGATTT-3′ | 5′-GTGCATCTCTTATGGCTTT-3′ | | 401bp |
| Oleastro, M. | Portuguese | 2010 | 5'-CCCCACAAGCGCTTAACAG-3' | 5'-GAGAGTGCCTAAACCCTATAATCC-3' | | Not mentioned |
| Schmidt, H. M. | Chinese | 2010 | 5'-CAAGCGCTTAACAGATAGGC-3' | 5'-AAGGCGTTTTCTGCTGAAGC-3' | | 450bp |
| Chiarini, A. | Italian | 2009 | 5'-CAAGCGCTTAACAGATAGGC-3' | 5'-AAGGCGTTTTCTGCTGAAGC-3' | | 450bp |
| LI, N. | Chinese | 2009 | Not mentioned | | Not mentioned | Not mentioned |
| Oleastro, M. | Portuguese | 2008 | 5'-CCATGAAAAAAGCTCTCTTACT-3' | 5'-GCCCTTTTACCCTTCGTTCAAC-3' | | Not mentioned |
|  | Portuguese | 2008 | 5'-CCATGAAAAAAGCTCTCTTACT-3' | 5'-GCCCTTTTACCCTTCGTTCAAC-3' | | Not mentioned |
| Yamaoka, Y.^a^ | Colombian | 2006 | NA | NA | | NA |
|  | American | 2006 | NA | NA | | NA |
| de Jonge, R. | Dutch | 2004 | 5'-CAAGCGCTTAACAGATAGGC-3' | 5'-AAGGCGTTTTCTGCTGAAGC-3' | | Not mentioned |
| Zambon, C. F. | Italian | 2003 | 5'-CAAGCGCTTAACAGATAGGC-3' | 5'-GCTTCACGAGAAAACGCCTT-3' | | Not mentioned |
| Yamaoka, Y. | American | 2002 | 5'-CCATGAAAAAAGCTCTCTTACT-3' | 5'-GCCCTTTTACCCTTCGTTCAAC-3' | | Not mentioned |
|  | Colombian | 2002 | 5'-CCATGAAAAAAGCTCTCTTACT-3' | 5'-GCCCTTTTACCCTTCGTTCAAC-3' | | Not mentioned |
| Yamaoka, Y. | Japanese | 2000 | 5'-CAAGCGCTTAACAGATAGGC-3' | 5'-GCTTCACGAGAAAACGCCTT-3' | | Not mentioned |
| For *oipA* gene (presence/absence) studies | | |  |  | |  |
| Ji, C. W. | Chinese | 2011 | 5'-GTGGCGTTGGTTCTGTTC-3' | 5'-GTGCGACTCTTGACTTGATT-3' | | 307bp |
| Ben Mansour, K. | Tunisian | 2010 | 5'-GTTTTTGATGCATGGGATTT-3' | 5'-GTGCATCTCTTATGGCTTT-3' | | 401bp |
| Xie, J. | Chinese | 2010 | 5'-CAAGCGCTTAACAGATAGGC-3' | 5'-AAGGCGTTTTCTGCTGAAGC-3' | | 450bp |
| Dabiri, H. | Persians | 2009 | 5′-GAGAATTCCACGCTGAAAGGAATGGAT-3′ | 5′-GATCCTCGAGTCAATAAACGCTCACCACTCTTT-3′ | | Not mentioned |
|  | Turkish | 2009 | 5′-GAGAATTCCACGCTGAAAGGAATGGAT-3′ | 5′-GATCCTCGAGTCAATAAACGCTCACCACTCTTT-3′ | | Not mentioned |
|  | Kurds, Lurs, Afghanis, Arabs | 2009 | 5′-GAGAATTCCACGCTGAAAGGAATGGAT-3′ | 5′-GATCCTCGAGTCAATAAACGCTCACCACTCTTT-3′ | | Not mentioned |
| Zhou, M. | Chinese | 2009 | 5'-GCAAGTAACAATCTCACCCCTTTC-3′ | 5′-TATCGTATTCATCATCACCGCTCC-3′ | | 100bp |
| Salih, B. A. | Turkish | 2007 | 5'-GTTTTTGATGCATGGGATTT-3' | 5'-GTGCATCTCTTATGGCTTT-3' | | 401bp |
| Zhang, J. | Chinese | 2004 | 5'-TCCACGCTGAAAGGAATGG-3' | 5'-CCATTTCCTGCGAATCGG-3' | | 232bp |

^a^, immunoblot method was used for *oipA* gene on/off detection in this study, thus, no primer was needed.

Supplementary Table 2. Detailed information of the included studies

| Author | Population | Year | *H. pylori* source | Test method for *H. pylori* status | Control group diseases | Detection method for *oipA* status | Age |
| --- | --- | --- | --- | --- | --- | --- | --- |
| For *oipA* functional status (on/off) studies | | | | | | | |
| Markovska, R. | Bulgarian | 2011 | not mentioned | Rapid urease test, histology | Chronic gastritis | PCR-sequencing | Adult |
| Oleastro, M. | Portuguese | 2010 | not mentioned | Culture | Non-ulcer dyspepsia | PCR-sequencing | Children |
| Schmidt, H. M. | Chinese | 2010 | not mentioned | Culture | Functional dyspepsia | PCR-sequencing | Adult |
| Chiarini, A. | Itlian | 2009 | Antrum, body | Rapid urease test, histology | Chronic gastritis | PCR-sequencing | Adult |
| LI, N. | Chinese | 2009 | Antrum | Rapid urease test, histology | Superficial gastritis | PCR-sequencing | Adult |
| Oleastro, M. | Portuguese | 2008 | Antrum | Culture | Non-ulcer dyspepsia | PCR-sequencing | Adult |
|  | Portuguese | 2008 | Antrum | Culture | Non-ulcer dyspepsia | PCR-sequencing | Children |
| Yamaoka, Y. | Colombian | 2006 | Antrum, corpus | Histology | Gastritis only | Immunoblot | Adult |
|  | American | 2006 | Antrum, corpus | Histology | Gastritis only | Immunoblot | Adult |
| de Jonge, R. | Dutch | 2004 | Not mentioned | Culture, histology | Gastritis | PCR-sequencing | Adult |
| Zambon, C. F. | Itilian | 2003 | Antrum | Culture, histology | Gastritis | PCR-sequencing | Adult |
| Yamaoka, Y. | American | 2002 | Antrum, corpus | Histology | Gastritis without ulcer or GC | PCR-sequencing | Adult |
|  | Colombian | 2002 | Antrum, corpus | Histology | Gastritis without ulcer or GC | PCR-sequencing | Adult |
| Yamaoka, Y. | Japanese | 2000 | Antrum | Culture | Gastritis without ulcer or GC | PCR-sequencing | Adult |
| For *oipA* gene (positive/negative) studies | | | | | | | |
| Ji, C. W. | Chinese | 2011 | Antrum | Rapid urease test, PCR, histology | Chronic gastritis | PCR-electrophoresis | Adult |
| Ben Mansour, K. | Tunisian | 2010 | Antrum, fundus | Culture | Gastritis | PCR-electrophoresis | Adult |
| Xie, J. | Chinese | 2010 | Antrum | Rapid urease test, histology | Chronic superficial gastritis | PCR-electrophoresis | Adult |
| Dabiri, H. | Persians | 2009 | Antrum | Culture, histology | Non-ulcer dyspepsia | PCR-electrophoresis | Adult |
|  | Turkish | 2009 | Antrum | Culture, histology | Non-ulcer dyspepsia | PCR-electrophoresis | Adult |
|  | Kurds, Lurs, Afghanis, Arabs | 2009 | Antrum | Culture, histology | Non-ulcer dyspepsia | PCR-electrophoresis | Adult |
| Zhou, M. | Chinese | 2009 | Antrum, body | Rapid urease test, histology | Chronic gastritis | PCR-electrophoresis | Adult |
| Salih, B. A. | Turkish | 2007 | Antrum | Culture | Gastritis | PCR-electrophoresis | Adult |
| Zhang, J. | Chinese | 2004 | not mentioned | Rapid urease test, PCR, histology | Gastritis | PCR-electrophoresis | Adult |

Supplementary Table 3. Results of Newcastle – Ottawa scale (NOS) assessment for the included studies

| Author | Year | Selection | Comparability | | Exposure | | Total NOS star rating | | |
| --- | --- | --- | --- | --- | --- | --- | --- | --- | --- |
| For *oipA* functional status (on/off) studies | | | |  | |  | |  | |
| Markovska, R. | 2011 | 3 | 1 | | 2 | | 6 | | |
| Oleastro, M. | 2010 | 2 | 1 | | 2 | | 5 | | |
| Schmidt, H. M. | 2010 | 2 | 1 | | 2 | | 5 | | |
| Chiarini, A. | 2009 | 2 | 1 | | 2 | | 5 | | |
| LI, N. | 2009 | 2 | 1 | | 1 | | 4 | | |
| Oleastro, M. | 2008 | 2 | 1 | | 1 | | 4 | | |
| Yamaoka, Y. | 2006 | 2 | 2 | | 2 | | 6 | | |
| de Jonge, R. | 2004 | 2 | 1 | | 2 | | 5 | | |
| Zambon, C. F. | 2003 | 3 | 1 | | 2 | | 6 | | |
| Yamaoka, Y. | 2002 | 2 | 2 | | 2 | | 6 | | |
| Yamaoka, Y. | 2000 | 2 | 1 | | 2 | | 5 | | |
| For *oipA* gene (positive/negative) studies | | | | | | | | |  |
| Ji, C. W. | 2011 | 3 | 1 | | 2 | | 6 | | |
| Ben Mansour, K. | 2010 | 3 | 1 | | 2 | | 6 | | |
| Xie, J. | 2010 | 3 | 1 | | 2 | | 6 | | |
| Dabiri, H. | 2009 | 3 | 1 | | 2 | | 6 | | |
| Zhou, M. | 2009 | 3 | 1 | | 2 | | 6 | | |
| Salih, B. A. | 2007 | 1 | 1 | | 2 | | 4 | | |
| Zhang, J. | 2004 | 2 | 1 | | 2 | | 5 | | |
