# Supplementary figures and images for: Association of presence/absence and on/off patterns of Helicobacter pylori oipA gene with peptic ulcer disease and gastric cancer risks: a meta-analysis
Source: BMC Infect Dis. 2013 Nov 20;13:555. doi: 10.1186/1471-2334-13-555 (PMC4225565; doi:10.1186/1471-2334-13-555)

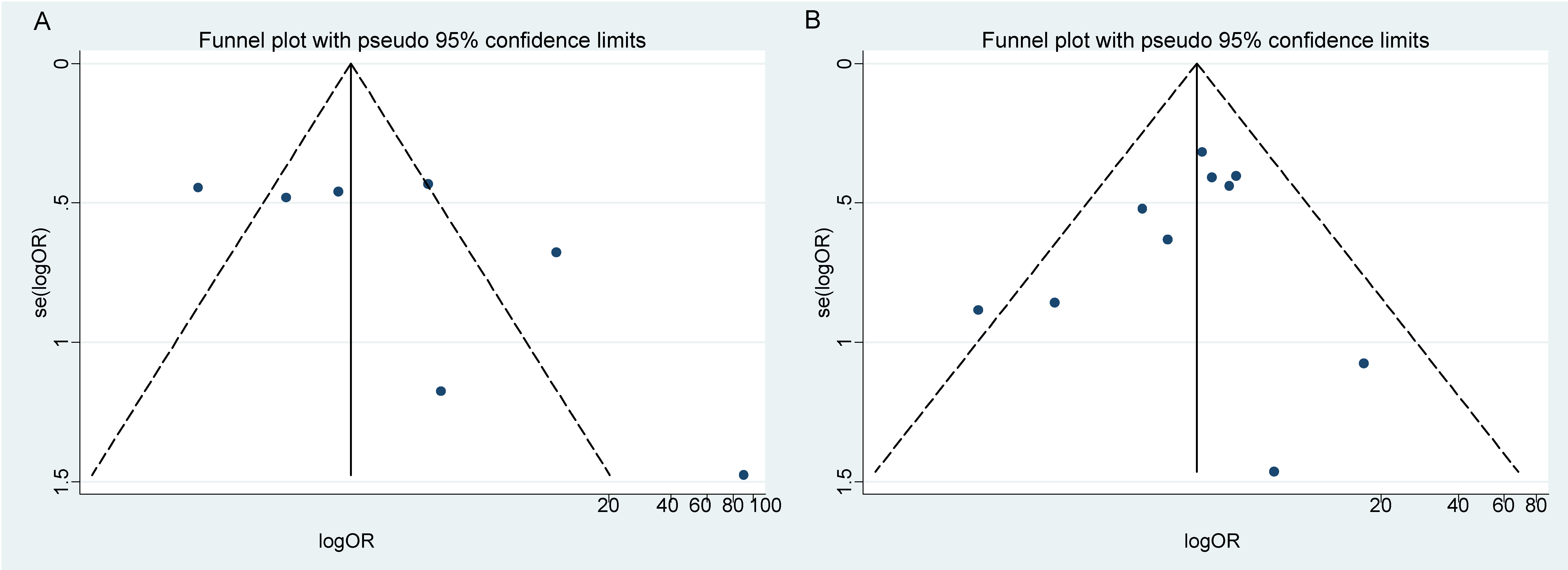

Supplement: Additional file 3: Figure S1 — Funnel plots of oipA gene presence/absence and oipA gene on/off status and PUD A, funnel plot for studies of association between oipA gene presence/absence and PUD; B, funnel plot for studies of association between oipA gene on/off status and PUD. [file 1471-2334-13-555-S3.tiff]
